# Revisiting Cytoreductive Nephrectomy in Metastatic Renal Cell Carcinoma: Real-World Evidence of Survival Benefit with First-Line Immunotherapy and Targeted Therapy Regimens

**DOI:** 10.3390/jcm14155543

**Published:** 2025-08-06

**Authors:** Sri Saran Manivasagam, Alireza Aminsharifi, Jay D. Raman

**Affiliations:** Department of Urology, Penn State College of Medicine and Milton S. Hershey Medical Center, 500 University Dr, Hershey, PA 17033, USA; smanivasagam@pennstatehealth.psu.edu (S.S.M.);

**Keywords:** metastatic renal cell carcinoma, cytoreductive nephrectomy, immunotherapy, targeted therapy

## Abstract

**Background**: Renal cell carcinoma (RCC) is a common malignancy with a rising global incidence. While cytoreductive nephrectomy (CRN) was historically a cornerstone in the management of metastatic RCC (mRCC), its role has been questioned following pivotal trials such as CARMENA and SURTIME. With the advent of immune checkpoint inhibitors (ICIs) and targeted therapies, the contemporary relevance of CRN coupled with first-line immunotherapy and targeted therapy combination regimens warrants re-evaluation. **Methods**: This retrospective cohort study utilized the TriNetX research network to identify patients aged 18–90 years diagnosed with mRCC between 2005 and 2024 who received first-line systemic therapies. Patients were stratified into two cohorts based on receipt of CRN status within one year of diagnosis. Propensity score matching (1:1) was done to adjust baseline characteristics. Kaplan–Meier survival analysis and Cox proportional hazards modeling were used to compare five-year overall survival between the groups. **Results**: Among 5960 eligible patients, 1776 (888 CRN matched to 888 who did not) formed the cohort of analysis. The CRN group demonstrated significantly higher five-year survival (57.7% vs. 45.0%, *p* < 0.0001) with a hazard ratio of 1.56 (95% CI: 1.33–1.83). Subgroup analyses showed consistent survival benefits across all four NCCN-recommended first-line regimens—Axitinib + Pembrolizumab: 64.0% (CRN) vs. 53.3% (no CRN), *p* = 0.01; Cabozantinib + Nivolumab: 50.1% vs. 40.4%, *p* = 0.004; Lenvatinib + Pembrolizumab: 37.4% vs. 22.8%, *p* = 0.012; Nivolumab + Ipilimumab: 56.4% vs. 46.1%, *p* = 0.005. **Conclusions**: In the era of modern immunotherapy and targeted agents, CRN remains associated with improved survival in patients with mRCC receiving NCCN-recommended first-line regimens. These findings support the continued evaluation of CRN as a component of multimodal therapy, particularly in patients with favorable risk profiles.

## 1. Introduction

Renal cell carcinoma (RCC) is a common genitourinary malignancy, with an estimated lifetime risk of 2.3% in men and 1.3% in women in the United States. Globally, RCC accounted for more than 430,000 new cases in 2022 [1,2]. The rising incidence of RCC is largely attributed to the increased incidental detection of renal masses through advanced imaging modalities [3,4]. While the majority of RCC cases are localized at diagnosis, metastatic disease is observed in approximately 11% to 33% of patients and is associated with significantly worse prognosis and reduced survival outcomes [5,6].

Management of metastatic RCC (mRCC) remains complex and lacks universally accepted treatment criteria. Cytoreductive nephrectomy (CRN)—the surgical removal of the primary renal tumor in the presence of distant metastases—was historically a mainstay of treatment. However, findings from pivotal trials such as the Cancer du Rein Metastatique Nephrectomie et Antiangiogéniques (CARMENA, 2018) and Immediate Surgery or Surgery After Sunitinib Malate in Treating Patients With Metastatic Kidney Cancer (SURTIME, 2019) have challenged its routine use [7,8]. For example, the CARMENA trial, which enrolled 450 patients with mRCC, demonstrated that treatment with sunitinib alone was not inferior to CRN followed by sunitinib, with median overall survival of 18.0 months versus 13.9 months, respectively [9].

Unlike many other malignancies, mRCC is largely resistant to cytotoxic chemotherapy. Current systemic therapies include targeted agents such as tyrosine kinase inhibitors (TKIs), vascular endothelial growth factor receptor (VEGFR) inhibitors, and immune checkpoint inhibitors (ICIs). ICIs target key regulatory pathways including programmed cell death protein 1 (PD-1), its ligands PD-L1 and PD-L2, and cytotoxic T-lymphocyte-associated protein 4 (CTLA-4), thereby enhancing CD8+ T-cell-mediated cytotoxicity against tumor cells [10].

Notably, both CARMENA and SURTIME trials were conducted prior to the widespread adoption of ICIs in the treatment of RCC. Therefore, the contemporary role of CRN in the era of modern immunotherapy remains an area of active investigation [7]. Select patients—particularly those with favorable risk profiles and limited metastatic burden—may still derive benefit from surgical intervention. The International Metastatic RCC Database Consortium (IMDC) criteria provide a validated framework for risk stratification, incorporating six prognostic factors: anemia, neutrophilia, thrombocytosis, hypercalcemia, poor performance status (Karnofsky score <80%), and a time interval of less than one year from diagnosis to initiation of systemic therapy [11].

The objective of this study is to evaluate survival outcomes in patients with mRCC who received National Comprehensive Cancer Network (NCCN)-recommended first-line systemic therapies (ICI-TKI combination regimens—Axitinib and Pembrolizumab; Cabozantanib and Nivolumb; Lenvatinib and Pembrolizumab; and ICI–ICI combination regimen: Nivolumab and Ipilimumab), comparing those who underwent CRN to those who did not. By leveraging a large, real-world population database, this study aims to clarify the contemporary role of CRN in the management of mRCC and potentially reinvigorate interest in its selective application to improve patient outcomes.

## 2. Materials and Methods

This retrospective cohort study was conducted using data from the TriNetX United States Collaborative research network, comprising 47 healthcare organizations and over 73 million patients globally. Patients were identified using ICD-10 diagnostic codes and CPT procedural codes. We used TriNetX, a global federated health research network providing access to electronic medical records (diagnoses, procedures, medications, laboratory values, genomic information) from large healthcare organizations. The TriNetX platform only uses aggregated counts and statistical summaries of de-identified information. No protected health information (PHI) or personal data is made available to the users of the platform. The study population included individuals aged 18 to 90 years diagnosed with mRCC who received National Comprehensive Cancer Network (NCCN)-recommended first-line systemic therapies (ICI-TKI combination regimens—Axitinib and Pembrolizumab; Cabozantanib and Nivolumb; Lenvatinib and Pembrolizumab; and ICI–ICI combination regimen: Nivolumab and Ipilimumab) between 2005 and 2024. The rates of mRCC patients who underwent CRN were calculated and tabulated. Patients were stratified into two cohorts: those who underwent CRN within one year of mRCC diagnosis and those who did not.

To minimize confounding, 1:1 propensity score matching (PSM) was performed using the TriNetX patient selection algorithm. Matching was based on demographic variables (age at index, gender, race, ethnicity), body mass index, comorbidities (hypertensive disorder, ischemic heart diseases, liver diseases, chronic lower respiratory tract diseases, chronic kidney disease, cerebrovascular disorders, diabetes mellitus, obesity and tobacco use) and lab parameters (creatinine, hemoglobin, leucocyte count, platelet count, serum calcium). Cohorts were randomly shuffled prior to matching. Baseline characteristics before and after matching are presented in Table 1 and Table 2.

Kaplan–Meier survival analysis was conducted to estimate and compare five-year overall survival across the groups. Survival distributions were compared using the log-rank test. Hazard ratios (HR) with 95% confidence intervals were calculated using Cox models. Proportional hazards assumptions were tested. All statistical analyses were performed using TriNetX’s built-in analytics platform. The primary outcome was five-year overall survival, defined as the time from initiation of first-line therapy to death from any cause.

## 3. Results

### 3.1. Temporal Trends in CRN from 2005 to 2024

**Figure 1:**
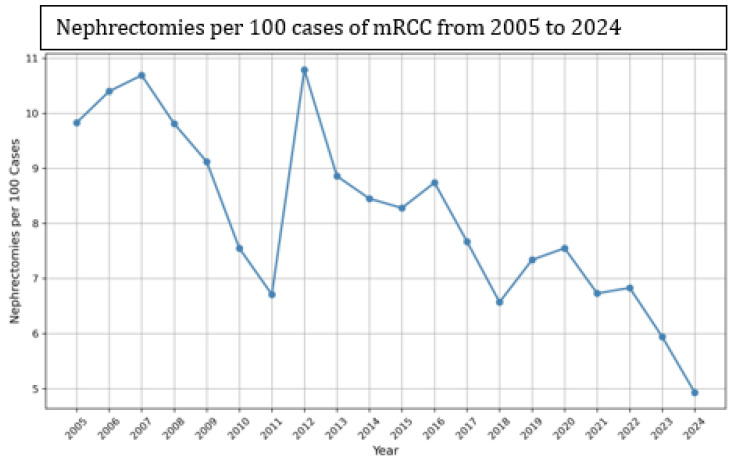
Trend in nephrectomies per 100 cases of metastatic renal cell carcinoma (mRCC) from 2005 to 2024.

Between 2005 and 2024, the rate of CRN per 100 cases of mRCC fluctuated, with the highest rate of 10.79 in 2012 and the lowest rate of 4.93 in 2024 (Figure 1). Between 2005 and 2012, the CRN rates fluctuated between 6.71 and 10.79. From 2012 onward, a consistent year-over-year decline was noted. This trend reflects a gradual reduction in CRN rates in the modern therapeutic era (Figure 1).

### 3.2. Baseline Characteristics

A total of 5960 patients with mRCC patients who received four types of first-line NCCN-recommended systematic therapies (Axitinib and Pembrolizumab, Cabozantanib and Nivolumab, Lenvatinib and Pembrolizumab, Nivolumab and Ipilimumab) were identified. Among these, 888 patients underwent CRN within one year of diagnosis. Propensity score matching (1:1) was conducted to compare this subgroup to 888 patients without CRN (total *n* = 1776).

Prior to propensity matching, patients who underwent CRN were younger (61.5 years vs. 63.8 years, *p*-value < 0.0001) and a lower proportion were Black (9.1% vs. 10.7%, *p* = 0.01). CRN patients also had lower BMI (29.1 vs. 30 kg/m^2^, *p* = 0.001), lower hemoglobin (12.2 vs. 12.7, *p* < 0.0001), higher platelet counts (295,000 vs. 268,0000, *p* < 0.0001), and lower creatinine (1.08 vs. 1.35, *p* < 0.0001). Interestingly, those patients who underwent CRN had higher rates of hypertension (62.2% vs. 53.9%, *p* < 0.0001), ischemic heart disease (22.5% vs. 18.5%, *p* = 0.005), liver diseases (20.3% vs. 16.5%, *p* = 0.005), and chronic lower respiratory tract diseases (20% vs. 16.9%, *p* = 0.02). The prevalence of chronic kidney disease was more common in those who did not undergo CRN (22.4% vs. 16.8%, *p* = 0.0002). The two cohorts did not differ in terms of prevalence of cerebrovascular disorders, diabetes, obesity, and tobacco use (Table 1). After propensity matching, the two cohorts were homogenous in terms of age, demographics and comorbidities. Despite propensity matching, the differences between the groups in terms of body mass index, hemoglobin, platelet counts, and creatinine, was still maintained. The characteristics of patients after matching have been summarized in Table 2.

### 3.3. Survival Analysis

**Figure 2:**
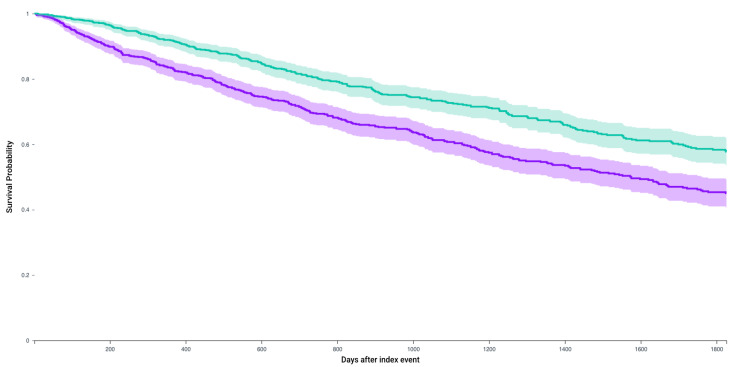
Kaplan–Meier survival analysis curves of patients who received any of the first-line regimens and those who did not receive CRN (purple) versus those who underwent CRN (green). Survival probability: 45.0% vs. 57.7%, hazard ratio 1.56, 95% CI = 1.33–1.83, *p* < 0.0001.

**Figure 3:**
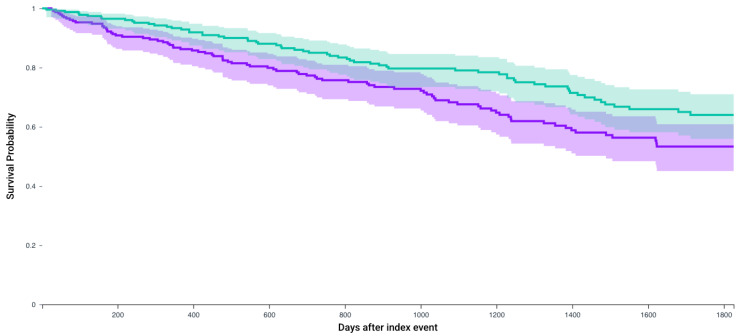
Kaplan–Meier survival analysis curves of patients who received Axitinib and Pembrolizumab and those who did not receive CRN (purple) versus those who underwent CRN (green). Survival probability 53.3% vs. 64%, hazard ratio 1.51, 95% CI = 1.08–2.09, *p*= 0.01.

**Figure 4:**
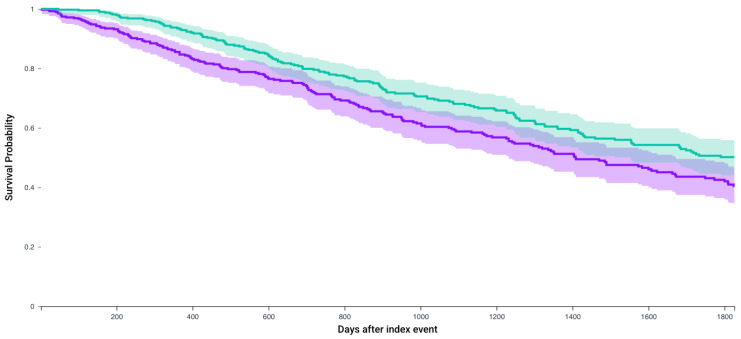
Kaplan–Meier survival analysis curves of patients who received Cabozantanib and Nivolumab and those who did not receive CRN (purple) versus those who underwent CRN (green). Survival probability 40.4% vs. 50.1%, hazard ratio 1.37, 95% CI = 1.10–1.71, *p*= 0.004.

**Figure 5:**
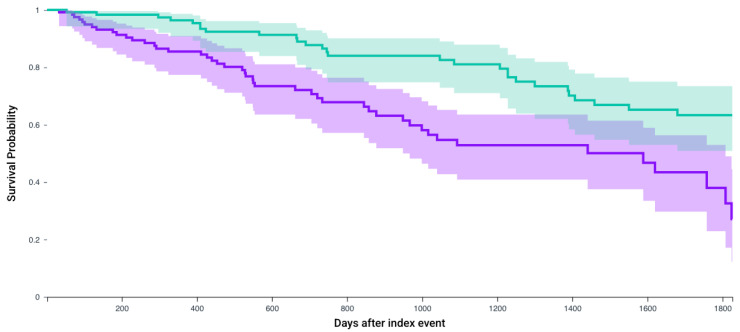
Kaplan–Meier survival analysis curves of patients who received Lenvatinib and Pembrolizumab and those who did not receive CRN (purple) versus those who underwent CRN (green). Survival probability 37.4% vs. 22.8%, hazard ratio 2.50, 95% CI = 1.55–4.03, *p*= 0.012.

**Figure 6:**
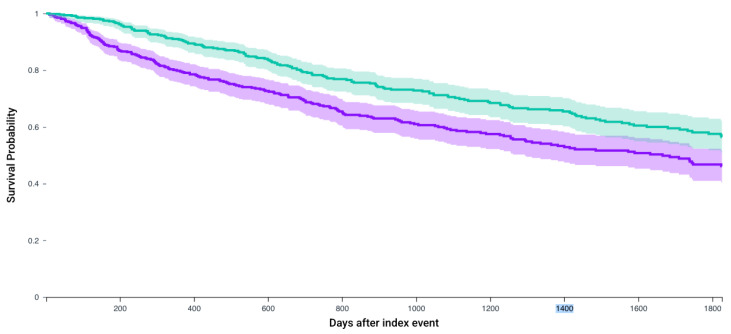
Kaplan–Meier survival analysis curves of patients who received Nivolumab and Ipilimumab and those who did not receive CRN (purple) versus those who underwent CRN (green). Survival probability 46.1% vs. 56.4%, hazard ratio 1.51, 95% CI = 1.22–1.87, *p*= 0.005.

Following matching, Kaplan–Meier analysis compared 5-year survival between patients who received any of the first-line 4 systemic therapy combinations and received CRN and those who did not receive CRN (Table 3). The survival probability was significantly higher in patients who received CRN (57.7% vs. 45.0%, *p* < 0.0001) with a significant hazard ratio (1.56, 95% CI = 1.33–1.83) (Figure 2). Similarly, for patients who receive Axitinib and Pembrolizumab after matching 235 patients, survival probability was higher in those who received CRN (64% vs. 53.3%, *p* = 0.01, Hazard ratio 1.51, 1.08–2.09) (Figure 3). This observation held true for patients receiving any of the first line recommended therapeutic combinations: Cabozantanib and Nivolumab (50.1% vs. 40.4%, *p* = 0.004, Hazard ratio: 1.37, 1.1–1.71) (Figure 4), Lenvatinib and Pembrolizumab (37.4% vs. 22.8%, *p* = 0.012, Hazard ratio: 2.5, 1.55–4.03) (Figure 5), and Nivolumab and Ipilimumab (56.4% vs. 46.1%, *p* = 0.005, Hazard ratio: 1.51, 1.22–1.87) (Figure 6).

## 4. Discussion

Cytoreductive nephrectomy (CRN) has long been a foundational component in the management of metastatic renal cell carcinoma (mRCC), particularly in the cytokine era, where its role was supported by both clinical rationale and survival data [12]. However, the emergence of targeted therapies and, more recently, immune checkpoint inhibitors (ICIs), has significantly altered the therapeutic landscape, prompting a re-evaluation of CRN’s utility [13]. Despite the evolving treatment paradigm, our study, based on real-world data from the TriNetX database, demonstrates a significant survival advantage in mRCC patients who underwent CRN compared to those who did not, even after rigorous propensity score matching. These findings challenge the prevailing trend of declining CRN utilization (10.79 per 100 cases of mRCC in 2012 to 4.93 per 100 cases of mRCC in 2024) and underscore the need for a nuanced, individualized approach to surgical intervention in mRCC.

The rationale for CRN historically rested on three pillars: symptom palliation, immunomodulation, and survival benefit. CRN was shown to alleviate local symptoms such as pain and hematuria, as well as paraneoplastic syndromes including hypertension, hypercalcemia, anemia, and polycythemia. From an immunological perspective, resection of the primary tumor was believed to reduce systemic immunosuppression and tumor burden, potentially enhancing the efficacy of systemic therapies and even triggering the abscopal effect, wherein distant metastases regress following removal of the primary tumor. Early clinical trials in the cytokine era supported these hypotheses, demonstrating improved survival in patients undergoing CRN [14,15,16,17].

However, the advent of VEGFR-targeted therapies prompted a reassessment of CRN’s role. Two pivotal randomized controlled trials, CARMENA and SURTIME, were launched to address this question. The CARMENA trial, a phase III non-inferiority study, compared immediate CRN followed by sunitinib with sunitinib alone in patients with mRCC. The trial concluded that sunitinib alone was non-inferior, with median overall survival (OS) of 18.4 months versus 13.9 months in the CRN group. However, the study had several limitations, including slow accrual, a high proportion of poor-risk patients, and potential selection bias, as patients deemed good surgical candidates were not enrolled [9]. Similarly, the SURTIME trial investigated the timing of CRN relative to systemic therapy, comparing immediate CRN followed by sunitinib with deferred CRN after three cycles of sunitinib. Although underpowered due to poor accrual, the trial suggested improved OS in the deferred CRN group (32.4 vs. 15.0 months), supporting a strategy of initial systemic therapy to identify responders [8].

Together, these trials suggest that immediate CRN should not be routinely performed in patients requiring systemic therapy. However, they also highlight that certain subgroups—particularly those with limited disease or favorable risk—may still benefit from deferred CRN. Importantly, both trials were conducted in the VEGFR-TKI era, and their findings may not fully apply to the current immunotherapy-dominated landscape.

Our study provides new insights into this evolving debate by analyzing survival outcomes in a large, real-world cohort of mRCC patients using the TriNetX database. We observed a marked decline in CRN utilization over the study period, from 10.79 CN procedures per 100 mRCC cases in 2012 to 4.93 per 100 cases in 2024. This trend likely reflects the influence of the CARMENA and SURTIME trials, as well as shifting clinical guidelines. However, our data suggest that this decline may be premature or overly broad, as CRN continues to confer a survival benefit in appropriately selected patients.

Before propensity matching, patients who underwent CRN were generally younger, more likely to be female, and there was a lower proportion of Black men. They also had lower body mass index (BMI), hemoglobin, and creatinine levels, but higher platelet counts. Interestingly, CRN recipients had a higher prevalence of comorbidities such as hypertension, ischemic heart disease, liver disease, and chronic lower respiratory tract disease, while chronic kidney disease (CKD) was less common. No significant differences were observed in the prevalence of cerebrovascular disease, diabetes, obesity, or tobacco use. These findings suggest a complex interplay between patient selection and treatment decisions, where comorbidities and baseline characteristics influence the likelihood of receiving CRN.

Propensity score matching was employed to balance the cohorts based on demographic, clinical, and laboratory variables. While matching effectively reduced many baseline differences, residual imbalances persisted in hemoglobin, creatinine, platelet count, and BMI. Despite these limitations, post-matching analysis revealed a statistically significant survival advantage in patients who underwent CRN, reinforcing the potential value of surgical intervention in selected mRCC patients.

The transition from the VEGFR-TKI era to the immunotherapy era has introduced new complexities in the management of mRCC. Current guidelines now recommend ICI-ICI or ICI-TKI combinations as first-line therapy [18,19]. However, the applicability of CARMENA and SURTIME findings to the immunotherapy era remains uncertain. Currently, few randomized trails are underway exploring the role of CRN in patients of mRCC receiving checkpoint inhibitors. The NORDIC-SUN-Trial compares deferred CRN with no CRN in mRCC patients receiving checkpoint inhibitors and aims to identify relevant biomarkers for personalized renal cancer management [20]. Similarly, the SWOG S1931 (PROBE) is a phase III randomized trial that aims to evaluate the survival benefit with CRN in mRCC patients receiving one of the approved ICI based combinations: Ipilimumab and Nivolumab, Axitinib and Pembrolizumab, or Axitinib and Avelumab [21]. Retrospective studies suggest that CRN may still confer benefit in select patients, particularly those with low-volume disease, good performance status, or those with favorable response to systemic therapy. However, these studies are limited by selection bias and lack of standardized criteria for CRN candidacy [22,23,24,25].

Risk stratification remains essential in guiding CRN decisions. The Memorial Sloan Kettering Cancer Center (MSKCC) and International mRCC Database Consortium (IMDC) models are widely used, incorporating clinical and laboratory parameters such as time from diagnosis to treatment, hemoglobin levels, leukocyte and platelet counts, performance status, and calcium levels [11]. However, these models were developed in the VEGFR-TKI era, and their relevance in the immunotherapy era is uncertain. The CheckMate 214 trial demonstrated that IMDC risk groups still correlate with treatment outcomes in the immunotherapy era, with intermediate and poor-risk patients deriving significant overall survival benefit from Nivolumab–Ipilimumab compared to sunitinib [26].

Emerging evidence suggests that molecular and genomic biomarkers may enhance patient selection for CRN. For instance, PD-L1 expression has been associated with improved outcomes in patients treated with ICIs. Genomic profiling may also identify patients more likely to benefit from CRN, although prospective validation is needed. A genomic or molecular profile might not only predict treatment response but also help identify patients who would derive the most benefit from surgical intervention [27,28].

Our study is the first to analyze survival outcomes in mRCC patients receiving standard first-line regimens, comparing those who underwent CRN with those who did not. The use of a large, real-world dataset and propensity score matching enhances the robustness of our findings. We observed significant differences in survival outcomes across individual systemic regimens, further establishing the efficacy of CRN. The observed trends in CRN utilization may reflect stringent patient selection based on existing risk stratification models and clinical judgment. While we attempted to match cohorts based on IMDC criteria, data regarding functional status (e.g., Karnofsky or ECOG scores) were lacking, which may influence the interpretation of our results.

This study offers several notable strengths that contribute to its relevance and impact in the current clinical landscape of metastatic renal cell carcinoma (mRCC). First and foremost, it represents one of the largest real-world analyses to date comparing survival outcomes between mRCC patients who underwent cytoreductive nephrectomy and those who did not, while receiving standard first-line systemic therapies. The use of the TriNetX global health research network enabled access to a diverse patient population, enhancing the generalizability of the findings across different healthcare settings.

A key methodological strength lies in the application of propensity score matching (PSM), which allowed for the creation of balanced cohorts by adjusting for a wide range of demographic, clinical, and laboratory variables. This statistical approach helps mitigate confounding and selection bias, which are inherent in observational studies. The inclusion of variables such as age, sex, comorbidities, and laboratory markers (e.g., hemoglobin, creatinine, platelet count) in the matching process strengthens the internal validity of the survival comparisons. Another strength is the study’s focus on real-world clinical practice, which complements the findings of randomized controlled trials (RCTs) such as CARMENA and SURTIME. Unlike RCTs, which often have strict inclusion criteria and may not reflect the broader patient population, real-world data offer insights into how CRN is applied in routine care and its impact on outcomes in a more heterogeneous cohort. This is particularly important given the underrepresentation of favorable-risk patients in prior RCTs and the lack of randomized data in the immunotherapy era.

Despite these strengths, the study is not without limitations. The retrospective, observational design inherently limits the ability to establish causality. While propensity matching reduces confounding, it cannot account for unmeasured variables, such as performance status (e.g., ECOG or Karnofsky scores), tumor burden, number and location of metastases, or patient preferences, all of which may influence both the decision to perform CRN and survival outcomes. The absence of these oncologic-specific variables in the TriNetX database restricts the granularity of the analysis and may introduce residual bias.

Another limitation is the reliance on administrative coding (ICD-10 and CPT codes) for patient identification, treatment classification, and outcome ascertainment. Coding inaccuracies, misclassification, or incomplete data entry may affect the reliability of the dataset. Additionally, the database does not capture cancer-specific mortality, which limits the ability to distinguish between deaths attributable to mRCC versus other causes. This is particularly relevant in a population with significant comorbidities, where non-cancer-related mortality may confound survival analyses.

The study also lacks detailed information on systemic therapy regimens, including dosing schedules, treatment duration, and adherence. Given the heterogeneity of first-line treatments in mRCC, especially in the IO era, this information would be valuable in interpreting the interaction between systemic therapy and CRN. Moreover, the absence of data on treatment response, progression-free survival, and quality of life outcomes further limits the comprehensiveness of the analysis. It is important to note that the patients with mRCC who received Lenvatinib and Pembrolizumab had overall survival probability of 22.8% in patients who did not receive CRN, as compared to 37.4% in patients who received CRN. It is noteworthy that among patients with metastatic renal cell carcinoma (mRCC) treated with Lenvatinib and Pembrolizumab, the overall survival probability was 22.8% in those who did not receive CRN, compared to 37.4% in those who did. In contrast, the CLEAR trial, which analyzed 355 patients receiving the same regimen, reported a 24-month overall survival rate of 79.2% [29]. The observed discrepancy in survival outcomes may be attributable to the smaller sample size in our cohort and should therefore be interpreted with caution.

Finally, while the study attempts to stratify patients based on IMDC risk criteria, the lack of data on functional status may compromise the accuracy of risk classification. This is particularly important given the central role of risk stratification in guiding CRN decisions. The inability to fully apply validated prognostic models underscores the need for more complete datasets and prospective studies that incorporate both clinical and molecular variables. It is important to note that patients who received CRN predominantly belonged to the IMDC favorable-risk group, whereas those in the non-CRN group were more likely to represent poor-risk categories. This imbalance in baseline risk profiles may partly explain the superior survival outcomes observed in the CRN group. These findings underscore the potential applicability of CRN in appropriately selected patients and highlight the importance of risk stratification when interpreting survival benefits.

In summary, while this study provides valuable real-world evidence supporting the survival benefit of CRN in selected mRCC patients, its findings should be interpreted in the context of its methodological limitations. Future prospective studies, ideally incorporating genomic and molecular biomarkers, are needed to refine patient selection and optimize the integration of CRN into contemporary treatment algorithms.

## 5. Conclusions

This real-world analysis showed a significant survival benefit of CRN in patients with mRCC treated with contemporary first-line systemic therapies. We demonstrated that despite a decline in utilization of CRN during the past two decades, CRN can be associated with an improved survival outcome in modern immunotherapy era and should be considered as part of a multidisciplinary treatment strategy. Future prospective trials with incorporation of several clinical data, tumor biology markers, modern immunotherapy regimens and CRN can optimize the treatment algorithms and CRN timing in patients with mRCC.

## Figures and Tables

**Table 1:** Baseline demographic and clinical information, lab parameters, and comorbidity profiles of mRCC patients who underwent CRN and those who did not, before propensity matching for variables. ICD-10 codes of comorbidities have been mentioned in the table wherever appropriate.

	Before Matching		
Parameter	No CRN 5072 cases	CRN 888 cases	*p*-value
Age at index, years Mean, SD	63.8, 11.1	61.5, 10.3	<0.0001
Gender Male Female	3529, 69.5% 1543, 30.4%	608, 68.4% 280, 31.5%	0.50 0.44
Race White Black Asian American Indian or Alaska Native Others	4177, 82.3% 546, 10.7% 273, 5.3% 52, 1.0% 24, 0.4%	714, 80.4% 81, 9.1% 55, 6.2% 20, 2.3% 18, 2.0%	0.18 0.01 0.42 0.24 0.17
Ethnicity Non-Hispanic/Latino Hispanic/Latino Unknown	3719, 73.3% 398, 7.8% 955, 18.8%	674, 75.9% 84, 9.5% 130, 14.6%	0.10 0.10 0.002
BMI [kg/m^2^], Mean, SD	30, 6.9	29.1, 6.2	0.001
Creatinine (Mass/volume), Mean, SD	1.35, 0.9	1.08, 0.5	<0.0001
Hemoglobin (Mass/volume), Mean, SD	12.7, 2.3	12.2, 2.3	<0.0001
Leukocyte count cells/mm^3^, Mean, SD	9820, 1910	7890, 3110	0.35
Platelet count in lakhs/mm^3^, Mean, SD	268, 111	295, 113	<0.0001
Calcium (Mass/volume), Mean, SD	9.39, 0.7	9.43, 0.7	0.15
HTN disease I10-I15	2738, 53.9%	552, 62.2%	<0.0001
Ischemic heart disease I20-25	941, 18.5%	200, 22.5%	0.005
Liver diseases, K70-77	837, 16.5%	180, 20.3%	0.005
Chronic lower respiratory tract diseases, J40-J4A	858, 16.9%	178, 20%	0.02
Chronic kidney disease, N18	1138, 22.4%	149, 16.7%	0.0002
Cerebrovascular disorders, I60-I69	430, 8.5%	87, 9.8%	0.19
Diabetes mellitus, E08-E13	1210, 23.9%	230, 26%	0.18
Overweight and obesity, E65-E68	1135, 22.4%	221, 24.8%	0.09
Tobacco use, Z72.0	251, 4.9%	44, 5%	0.99

**Table 2:** Baseline demographic, clinical, lab parameters, and comorbidity profiles of mRCC patients who underwent CRN and those who did not, after propensity matching for variables. ICD-10 codes of comorbidities have been mentioned in the table wherever appropriate.

	After Matching		
Parameter	No CRN 888 cases	CRN 888 cases	*p*-value
Age at index, years Mean, SD	62.2, 11.7	61.5, 10.3	0.17
Gender Male Female	648, 72.9% 240, 27%	646, 72.7% 242, 27.3%	0.91 0.91
Race White Black Asian American Indian or Alaska Native Others	726, 81.8% 96, 10.8% 32, 3.6% 10, 1.1% 24, 2.7%	714, 80.4% 81, 9.1% 55, 6.2% 20, 2.3% 18, 2%	0.46 0.28 0.7 0.48 0.85
Ethnicity Non-Hispanic/Latino Hispanic/Latino Unknown	697, 78.5% 73, 8.2% 118, 13.3%	674, 76% 84, 9.4% 130, 14.6%	0.19 0.35 0.41
BMI [kg/m^2^], Mean, SD	29.9, 7.0	29.1,6.2	0.01
Creatinine (Mass/volume), Mean, SD	1.27, 0.7	1.08, 0.5	0.0001
Hemoglobin (Mass/volume), Mean, SD	12.7, 2.3	12.2, 2.3	0.0002
Leukocyte count cells/mm^3^, Mean, SD	8820, 2410	7890, 3110	0.38
Platelet count in lakhs/mm^3^, Mean, SD	266,107	295, 113	<0.0001
Calcium (Mass/volume), Mean, SD	9.39, 0.9	9.43, 0.7	0.38
HTN disease I10-I15	554, 62.4%	552, 62.2%	0.92
Ischemic heart disease I20-25	184, 20.7%	200, 22.5%	0.35
Liver diseases, K70-77	161, 18.1%	180, 20.3%	0.25
Chronic lower respiratory tract diseases, J40-J4A	181, 20.4%	178, 20.1%	0.86
Chronic kidney disease, N18	153, 17.2%	149, 16.8%	0.80
Cerebrovascular disorders, I60-I69	88, 9.9%	87, 9.8%	0.93
Diabetes Mellitus, E08-E13	221, 25.1%	230, 25.9%	0.62
Overweight and obesity, E65-E68	212, 23.8%	221, 24.8%	0.62
Tobacco use, Z72.0	42, 4.7%	44, 5.0%	0.82

**Table 3:** Summary of Kaplan–Meier survival analysis for mRCC patients receiving different first-line systemic therapy regimens. Each subcohort has been individually matched and therefore the number in each subcohort will not add up to 888.

	N, In Each Cohort	No CRN Survival Probability, % (Mean Follow-Up in Days)	CRN Survival Probability, % (Mean Follow-Up in Days)	Hazard Ratio, 95% CI	*p*-Value
Patients who receive any of the first-line regimens	888	45.0% (729.5 days)	57.7% (887 days)	1.56 (1.33–1.83)	<0.0001
Patients who receive Axitinib and Pembrolizumab	235	53.3% (743.4 days)	64% (857.3 days)	1.51 (1.08–2.09)	0.01
Patients who receive Cabozantanib and Nivolumab	387	40.4% (642.1 days)	50.1% (672 days)	1.37 (1.10–1.71)	0.004
Patients who receive Lenvatinib and Pembrolizumab	123	22.8% (470.9 days)	37.4% (561.5 days)	2.50 (1.55–4.03)	0.012
Patients who receive Nivolumab and Ipilimumab	479	46.1% (748.4 days)	56.4% (844.2 days)	1.51 (1.22–1.87)	0.005

## Data Availability

The data has been obtained from TriNetX and is only available to participating institutions.

## Abbreviations

The following abbreviations are used in this manuscript:

RCC	Renal Cell Carcinoma
mRCC	Metastatic Renal Cell Carcinoma
ICI	Immune Checkpoint Inhibitors
CRN	Cytoreductive Nephrectomy
CARMENA	Cancer du Rein Metastatique Nephrectomie et Antiangiogéniques
SURTIME	Immediate Surgery or Surgery After Sunitinib Malate in Treating Patients With Metastatic Kidney Cancer
PD-1	Programmed cell death protein 1
PD-L1 and PD-L2	Programmed cell death protein ligand 1 and 2
CTLA4	Cytotoxic T-lymphocyte associated protein 4
NCCN	National Comprehensive Cancer Network
TKI	Tyrosine Kinase Inhibitors
VEGFR	Vascular Endothelial Growth Factor Receptor Inhibitors
IMDC	International Metastatic RCC Database Consortium
BMI	Body Mass Index
HTN	Hypertension
